# Preclinical and clinical evaluation through serial colonoscopic evaluation of neratinib‐induced diarrhea in HER2‐positive breast cancer—A pilot study

**DOI:** 10.14814/phy2.70008

**Published:** 2024-08-26

**Authors:** Joanne Bowen, Sofia Braga, Valeria Dal Zotto, John Finnie, Daniel DiPrimeo, Blaire Cooke, Georg F. Bischof, Alvin Wong, Jack A. Di Palma

**Affiliations:** ^1^ School of Biomedicine The University of Adelaide Adelaide South Australia Australia; ^2^ Medical Oncology Hospital Prof. Doutor Fernando Fonseca, EPE Amadora Portugal; ^3^ Department of Pathology The University of Alabama at Birmingham Birmingham Alabama USA; ^4^ Division of Research and Innovation University of Adelaide Adelaide South Australia Australia; ^5^ Puma Biotechnology, Inc. Los Angeles California USA; ^6^ Division of Gastroenterology University of South Alabama, College of Medicine Mobile Alabama USA

**Keywords:** colonoscopy study, diarrhea, HER2‐positive breast cancer, neratinib, rat model

## Abstract

The irreversible pan‐HER tyrosine kinase inhibitor neratinib is approved for patients with HER2‐positive, early‐stage and metastatic breast cancer (BC). Neratinib‐associated diarrhea is the most common reason for early discontinuation. Preclinical studies identified mechanisms of neratinib‐induced diarrhea and rationale for prophylactic and preventive measures. We studied effects of neratinib on rat intestines and conducted a phase 2 study of colon pathogenesis in patients with HER2‐positive BC treated with neratinib (NCT04366713). Colon samples from female albino Wistar rats receiving neratinib or vehicle were examined for histopathological changes. Patients with HER2‐positive BC received neratinib 240 mg once daily for up to 1 year. Colonoscopy biopsies were collected at baseline and at Day 28 to identify changes consistent with rat pathologies. Rat colons were markedly altered in appearance, with similar short circuit currents (*I*_sc_) and responses to carbachol and forskolin. Mucosal barrier loss and/or significant increase in secretory propensity in neratinib‐ versus control‐treated animals were not seen. Two of four endpoint‐evaluable patients presented with mild pathological changes, largely comparable with the rat model. Preclinical evidence supports an inflammatory component of neratinib‐induced diarrhea without mucosal barrier function loss. Colonoscopy findings in patients with BC indicate mild or no pathological changes in the colon due to neratinib treatment.

## 1 INTRODUCTION

Neratinib is an irreversible pan‐HER tyrosine kinase inhibitor (TKI) approved in 2017 by the US Food & Drug Administration as monotherapy for the extended adjuvant treatment of patients with HER2‐positive early‐stage breast cancer (EBC) following adjuvant trastuzumab‐based therapy and in combination with capecitabine for patients with HER2‐positive metastatic breast cancer (MBC) (Food & Drug Administration, 2022). In the European Union, neratinib was approved in 2018 as extended adjuvant treatment of adult patients with early‐stage hormone receptor‐positive HER2‐positive breast cancer (BC) who completed adjuvant trastuzumab‐based therapy less than 1 year previously (European Medicines Agency, 2022).

Diarrhea, which is commonly observed in patients undergoing treatment with TKIs (Le Du et al., 2021; Secombe et al., 2020), is the most frequently reported adverse event (AE) associated with neratinib, particularly in the absence of antidiarrheal prophylactic treatment (Awada et al., 2016; Chan et al., 2016; Mortimer et al., 2019; Saura et al., 2019). In the ExteNET trial (NCT00878709) of neratinib following adjuvant trastuzumab, in which no mandatory antidiarrheal prophylaxis was used, 40% of neratinib‐treated patients reported grade 3 diarrhea, and 17% of patients discontinued neratinib as a result of diarrhea (Chan et al., 2016; Martin et al., 2017). Multiple antidiarrheal strategies have been evaluated since the ExteNET study, and 2‐week dose escalation of neratinib as well as loperamide prophylaxis are now included in the United States prescribing information for neratinib and in the NCCN Breast Cancer Clinical Guidelines v4.2024 (Barcenas et al., 2020; Chan et al., 2023; Food & Drug Administration, 2022; National Comprehensive Cancer Network, 2024).

To investigate the underlying mechanisms of neratinib‐induced diarrhea, rat models of neratinib‐related diarrhea have been developed (Bowen et al., 2012; Secombe et al., 2019, 2021). These models identified inflammation of the terminal ileum and proximal colon as one potential factor in neratinib‐related diarrhea: for example, neratinib significantly increased levels of pro‐inflammatory IFN‐ɣ in the ileum, and inflammatory infiltrate was seen in the intestines of neratinib‐treated rats (Secombe et al., 2019). Rats treated with neratinib presented with higher levels of apoptosis in the proximal colon compared with control animals (Secombe et al., 2019). Moreover, data from these models showed that neratinib‐mediated diarrhea pathogenesis also included anatomical disruption, characterized by blunting and fusion of villi in the ileum as well as a disruption and shortening of crypts in the colon (Secombe et al., 2021). Interestingly, the ileum showed the most enunciated histopathological changes, and it has been postulated that any effects of neratinib might be most remarkable in that area due to the fact that ErbB1 (EGFR)—a target of neratinib—is expressed at relatively high levels in that location compared with the rest of the gastrointestinal tract (Secombe et al., 2019, 2021; Van Sebille et al., 2017).

We now report the results of preclinical and clinical studies in which we evaluated pathophysiological changes to the colon following neratinib treatment. First, we examined colon histopathology and permeability in neratinib‐treated rats using the previously reported rat model of diarrhea (Bowen et al., 2012; Secombe et al., 2021) to assess if the observed changes were consistent with previously described findings. Second, we undertook an open‐label, phase 2 study of colon pathology via colonoscopy‐based imaging and biopsies in patients receiving neratinib for HER2‐positive BC to examine if any pathological changes observed in patients were concordant with the observations made in the rodent model.

## 2 METHODS

### 2.1 Preclinical study

Preclinical experiments were conducted on female albino Wistar rats obtained from the Animal Resource Centre, Perth, Australia. Rats were housed in groups of four or five in individually ventilated cages. Temperature was maintained between 19°C and 23°C and relative humidity within the range of 45%–65% with a 12‐h light/dark cycle. Food (standard laboratory rat chow; Specialty Feeds, Australia) and water were consumed ad libitum. Any rats experiencing moderate to severe treatment‐related toxicity (e.g., diarrhea, weight loss, stress marks) were allowed soaked chow (normal feed softened in water to ease mastication). Rats were acclimatized to local housing conditions for a minimum of 7 days prior to the first day of dosing. On Day 1 of treatment, the rats were between 7 and 9 weeks old.

This study was approved by the Animal Ethics Committee of the University of Adelaide (study number M‐2019‐025) and complied with the National Health and Medical Research Council (Australia) Code of Practice for Animal Care in Research and Training (2013).

The conducting laboratory, not the study sponsor, has ownership of the data associated with the performed preclinical experiments.

#### 2.1.1 Experimental design

Rats were randomly assigned to vehicle control (0.5% w/w hydroxypropyl methylcellulose buffer; *n* = 6; purchased from Sigma‐Aldrich) or neratinib (50 mg/kg; *n* = 6; supplied by Puma Biotechnology, Inc., batch no. 1411804) based on a prior study that showed this to be a dose at which diarrhea was considered reproducible and clinically relevant (Secombe et al., 2021). During the 28‐day neratinib treatment period, rats received daily oral gavages using a soft plastic feeding tube coated in 30% sucrose solution. Neratinib or vehicle were given at a constant dose volume of approximately 5 mL/kg. Individual dose volumes were adjusted daily according to the body weight of each rat on each treatment day. The first day of dosing was designated Day 1. The final dose was given on the day before the scheduled necropsy. All rats were deeply anesthetized via isoflurane inhalation and culled by cardiac exsanguination, with death confirmed by cervical dislocation.

#### 2.1.2 Tissue collection, preparation, and examination

At necropsy, the gastrointestinal tract from the pyloric sphincter to the rectum was removed and flushed with chilled, sterile 1× phosphate buffered saline. Samples of colon (at 67% of length from the cecal–colon junction) were collected and fixed in 10% formalin for embedding in paraffin. Paraffin‐embedded intestinal samples were cut with a rotary microtome (RM2235, Leica) and 4 μm sections were mounted onto Superfrost glass slides (Menzel‐Glaser). Routine hematoxylin (Harris hematoxylin catalog no. AAH) and eosin (Australian Biostain, catalog no. AEAW) (H&E) staining was completed. Images of all stained slides were taken using a Nanozoomer digital slide scanner (Hamamatsu Photonics, Shizouka, Japan) and viewed using the Nanozoomer Digital Pathology Software (View v1.2, Histalim, Montpelier, France). All analysis was conducted in a blinded fashion by a specialist veterinarian pathologist.

#### 2.1.3 Mucosal barrier function

A small section of distal colon tissue was cut longitudinally along the mesentery and external muscle layers were carefully dissected and removed under microdissection microscopes in a Ringer's (NaCl 115.4; KCl 5; MgCl_2_ 1.2; NaH_2_PO_4_ 0.6; NaHCO_3_ 25; CaCl_2_ 1.2, and glucose 10 mmol/L)/indomethacin (1 μM solution; purchased from Sigma‐Aldrich) solution. Tissue was then mounted into Ussing chambers (Physiologic Instruments; EM‐CSYS‐8; Reno, NV, USA) and kept in conditions and analyzed as previously described (Van Sebille et al., 2018). Tissue was assessed for baseline short circuit current (*I*_sc*−*_ μA/cm^2^) and response to 100 μM carbachol (purchased from Sigma‐Aldrich; catalog no. C4382) or 10 μM forskolin (purchased from Sigma‐Aldrich; catalog no. F3917) (ΔμA/cm^2^) using Acquire and Analyze software 2.3 (Physiologic Instruments; Reno, NV, USA). Integrity of the colonic mucosal barrier was also measured by resistance (Ω/cm^2^) and permeability to 0.5 mg/mL 4 kDa fluorescein isothiocyanate (FITC)‐dextran (purchased from Sigma‐Aldrich; catalog no. FD4) added to the apical chamber. Concentration of FITC in the serosal chamber at 2 h was determined in triplicate using a fluorescent plate reader (BioTek Synergy, Winooski, VT, USA) against a standard curve (range 0.0001–10 μg/mL).

#### 2.1.4 Statistics

All comparisons of means were conducted using an unpaired, two‐tailed Student's *t*‐test in Prism v8.0 (GraphPad, Boston, MA, USA), with statistical significance set at *p* < 0.05.

### 2.2 Pilot clinical study design

This was an open‐label, phase 2 study investigating colon pathology in patients with HER2‐positive BC who were treated with neratinib monotherapy. Patients were aged 18 years or older at signing of informed consent, with Eastern Cooperative Oncology Group (ECOG) status of 0 or 1, histologically confirmed stage I−IV primary adenocarcinoma of the breast, and documented HER2 overexpression or amplification.

Patients with confirmed stage I to stage IIIc BC who were receiving neratinib for extended adjuvant treatment had to have completed a course of prior adjuvant trastuzumab or experienced side effects resulting in early discontinuation of trastuzumab that had resolved. Patients with MBC were required to have received at least two prior HER2‐directed regimens. Patients were not allowed to receive concurrent chemotherapy, radiotherapy, immunotherapy, or biotherapy for BC (stage I−IIIc disease) or prior capecitabine or HER2‐directed TKI therapy (MBC only).

This study was approved by the Ethics Committee for Clinical Research (CEIC) and the National Authority for Medicines and Health Products, I.P. (INFARMED) in Portugal (CEIC Code: 20190900, decision number 48/VPCD/2020). The study was carried out in accordance with the current revised version of the Declaration of Helsinki and Good Clinical Practice and other applicable legislation.

#### 2.2.1 Treatment and assessments

Patients with stage I–IIIc BC: neratinib (supplied by Puma Biotechnology, Inc.) in the extended adjuvant setting was administered at a single daily 240 mg dose until completion of 1 year of therapy, or until disease recurrence (as determined by the investigator), death, unacceptable toxicity, or other specified withdrawal criterion. For patients with MBC, neratinib (supplied by Puma Biotechnology, Inc.) was administered as above during cycle 1 (until Day 28); capecitabine (750 mg/m^2^ twice daily for 14 days of each 21‐day treatment cycle; generic capecitabine [Accord Healthcare] supplied by the site pharmacy) was introduced after the second colonoscopy procedure, with continuation of daily neratinib. All patients received diarrhea prophylaxis with loperamide (generic loperamide [Generis Farmaceutica] provided by the site pharmacy) on Days 1–28 during cycle 1, and as needed thereafter.

Two colonoscopies were performed: the first was done after confirmation of eligibility but before administration of the first neratinib dose and the second was done on day 30 ± 3 days, that is, at the conclusion of cycle 1. During colonoscopy, photographs of the colonic mucosa were taken, along with approximately eight biopsies from the terminal ileum, cecum, ascending colon, proximal transverse colon, splenic flexure, sigmoid colon, and rectum. Any colon mucosa that appeared to be abnormal was noted and biopsied in a separate jar. Biopsy material was analyzed in a central laboratory (H&E staining performed with Leica Hematoxylin and Eosin S3 staining kit on the Leica HistoCore Spectra ST stainer; Leica Biosystems, Deer Park, IL, USA). If polyps were identified, these were removed during the examination; if cancer was identified, this too was sampled.

At the time of colonoscopy, levels of serological inflammatory markers (erythrocyte sedimentation rate [ESR] and C‐reactive protein [CRP]) and stool markers for diarrhea (fecal calprotectin, fecal elastase) were assessed via routine blood and fecal testing panels.

The primary analysis was performed when all patients had completed two consecutive colonoscopies with biopsies. The final analysis was performed when all patients had completed study treatment, discontinued treatment, or withdrawn for other reasons. The study ended when all patients had been followed for 28 days after the last dose of neratinib. Clinical care of the patient beyond the scope of the study was the responsibility of the treating physician.

#### 2.2.2 Study objectives and endpoints

The primary objective was to characterize and understand colon pathogenesis related to neratinib‐induced diarrhea through biopsies and images obtained by colonoscopy. The primary endpoint was the change from baseline in pathological findings in colon biopsies after the first 28 days of neratinib monotherapy.

Secondary objectives were to characterize the incidence and severity of diarrhea during the first 28‐day cycle and analyze changes in serological and fecal inflammatory markers from baseline to the second colonoscopy. Patient response and recurrence data were maintained by the site prospectively but were not collected as part of the trial.

All patients who received neratinib were included in the safety analysis. The incidences of treatment‐emergent AEs and serious adverse events (SAEs) were summarized (1) during cycle 1 (through Day 28), (2) during the 28 days following the second colonoscopy in patients with stage I–IIIc BC, and (3) following the completion of cycle 2 (through Day 56) in patients with MBC after all biopsies were completed. Only SAEs were followed from cycle 2 until 28 days after neratinib was discontinued. AEs and SAEs were graded according to the National Cancer Institute Common Terminology Criteria (National Cancer Institute CTCAE), version 4.0.

#### 2.2.3 Statistical considerations

All clinical analyses were descriptive.

## 3 RESULTS

### 3.1 Preclinical study

#### 3.1.1 Histopathology

**FIGURE 1:**
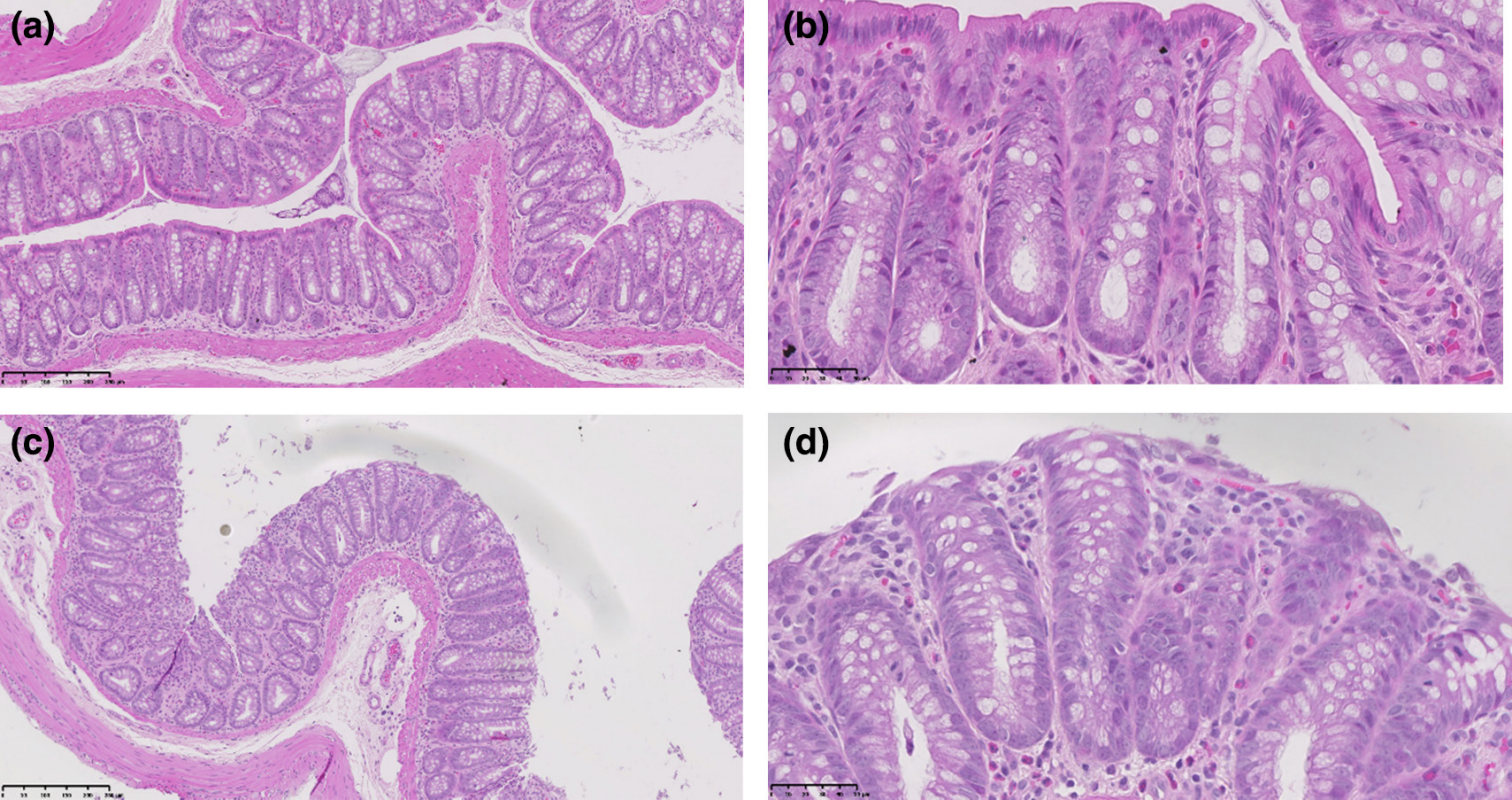
Intestinal histopathological injury. Representative images of hematoxylin and eosin staining in controls (a, b) and neratinib‐treated rats (c, d). Images show the distal colon at 10× (a, c) or 40× (b, d) magnification.

Colons from vehicle‐treated (*n* = 6) and neratinib‐treated (*n* = 6) animals were compared. In the distal third of colons from control animals, the tubular glands had a normal proliferative compartment (crypts of Lieberkühn) at their base and the upper portion was lined by absorptive, columnar epithelial cells (enterocytes) with basally located nuclei. Mucus‐secreting goblet cells were abundant, and there was a small inflammatory infiltrate in the lamina propria; submucosa comprised mainly lymphocytes and polymorphonuclear leukocytes, including eosinophils (Figure 1a,b).

Colons from neratinib‐treated rats displayed frequently damaged or markedly modified lumen‐lining surface enterocytes (Figure 1c,d). Epithelial degeneration and necrosis, with exfoliation of effete cells into the intestinal lumen, leaving a residual, denuded surface, was observed in some areas. More often, migration of adjacent, flattened, viable epithelial cells across the denuded luminal surface was observed. Some crypts there displayed marked epithelial proliferation, with less‐differentiated cells showing cytoplasmic basophilia. The mitotic index was increased, particularly in the proliferative compartment in the depth of the glands, but also in more superficial glandular regions. Very few apoptotic bodies were detected. Increased mucus extrusion over the mucosal surface was frequently observed. The inflammatory infiltrate in the lamina propria and submucosa was prominent and largely comprised of lymphocytes and polymorphonucleocytes, the latter being mainly eosinophils. Moreover, intraepithelial lymphocytes were often present.

#### 3.1.2 Mucosal barrier function

**FIGURE 2:**
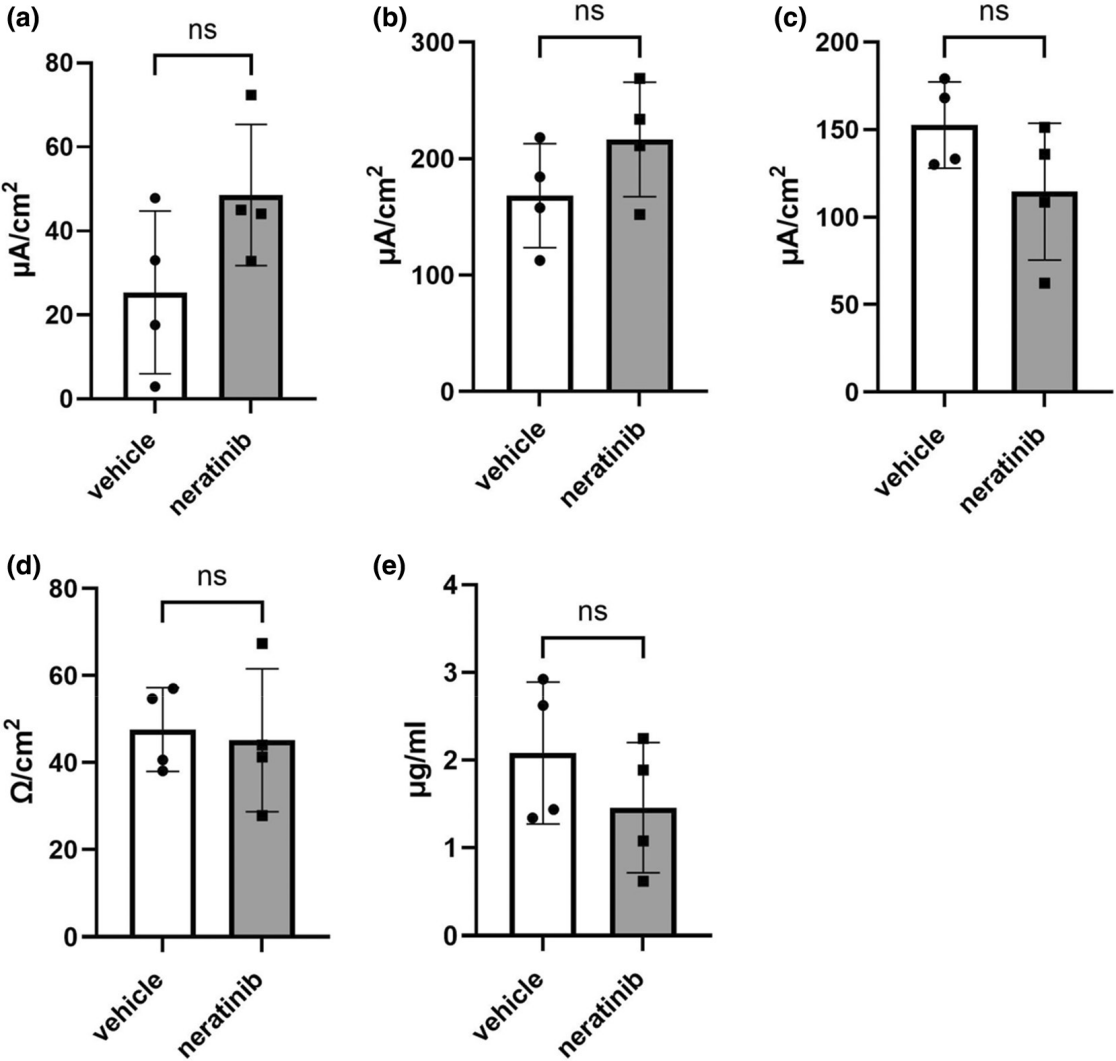
Colonic mucosal barrier function in vehicle‐ and neratinib‐treated rats: (a) baseline *I*_sc_; change in *I*_sc_ in response to secretagogues (b) forskolin and (c) carbachol; (d) baseline resistance; and (e) concentration of 4kD FITC‐dextran at 2 h. Data shown are means ± standard deviations; circles represent individual datapoints.

No significant differences were observed between vehicle‐treated (*n* = 4) and neratinib‐treated (*n* = 4) groups for any measure of mucosal barrier function, including baseline *I*_sc_ (Figure 2a), change in *I*_sc_ in response to secretagogues (Figure 2b,c), baseline resistance (Figure 2d), or concentration of FITC (Figure 2e). This indicates that the mucosal barrier remains intact and functional despite inflammatory infiltrate being present. It also shows the epithelial changes are compensatory, indicating that the histopathology was self‐resolving.

### 3.2 Pilot clinical study case presentations

Seven patients from a single study center provided informed consent for participation in the study, one of whom later withdrew. One patient was diagnosed with ulcerative colitis at the first colonoscopy and was excluded from the study; the safety population thus comprised five patients (Figure S1; CONSORT diagram). All patients were female; three were premenopausal and two were postmenopausal. One patient received neratinib in the extended adjuvant setting and the remaining four patients received neratinib for MBC. One patient (Patient 003) did not complete the second colonoscopy due to progressive disease (PD; grade 3 pleural effusion and grade 3 pericardial effusion on Day 21) and was thus not considered evaluable for the primary study endpoint. All four endpoint‐evaluable patients experienced at least one episode of diarrhea, either during cycle 1, after cycle 1 and beyond, or at multiple timepoints. All diarrhea episodes in endpoint‐evaluable patients were grade 1. During the screening consultation, patients were asked about their bowel movements to determine if they were constipation‐prone (CP) or diarrhea‐prone (DP). Patient characteristics are summarized in Table 1.

**TABLE 1:** Individual patient data (safety evaluable population).

	Patient 001		Patient 003		Patient 004		Patient 005		Patient 006	
Age	45		43		70		40		30	
Sex	Female		Female		Female		Female		Female	
Race	White		White		White		White		White	
Menopausal status	Postmenopausal		Premenopausal		Postmenopausal		Premenopausal		Premenopausal	
ECOG	0		1		0		0		0	
Prior anticancer treatments										
Neoadjuvant	Doxorubicin; paclitaxel; trastuzumab						Pertuzumab; tamoxifen; trastuzumab			
Adjuvant	Trastuzumab + goserelin + tamoxifen; trastuzumab + tamoxifen		Goserelin; tamoxifen; cyclophosphamide; PEG‐doxorubicin; paclitaxel; anastrozole		5‐Fluorouracil + epirubicin; docetaxel + trastuzumab; trastuzumab + letrozole		Docetaxel + carboplatin trastuzumab + pertuzumab; trastuzumab + pertuzumab; letrozole			
Metastatic/locally advanced	Docetaxel + pertuzumab + trastuzumab; T‐DM1		Exemestane; pertuzumab; trastuzumab; T‐DM1		Paclitaxel + pertuzumab + trastuzumab; maintenance pertuzumab + trastuzumab + anastrozole; T‐DM1; vinorelbine + trastuzumab				Docetaxel + pertuzumab + trastuzumab; goserelin; zoledronate; T‐DM1	
Diarrhea, maximal grade	1		1		1		1		1	
Laboratory tests	Day 0	Day 28	Day 0	Day 28	Day 0	Day 28^a^	Day 0	Day 28	Day 0	Day 28
CRP mg/L	<0.5	1.7	1.9	N/A	0.9	1	1.7	N/A	2.2	1.4
ESR mm/h	16	9	14	N/A	23	15	23	N/A	11	12
CALPRO μg/g	N/A	34	868	N/A	291	377	21	71	134	555
ELA1 μg/g	N/A	256	189	N/A	426	388	>500	>500	>500	464

Abbreviations: CALPRO, calprotectin (normal range: <5–50 μg/g); CRP, C‐reactive protein (normal: <9 mg/L); ECOG, Eastern Cooperative Oncology Group; ELA1, elastase (normal: >200 μg/g); ESR, erythrocyte sedimentation rate (normal values for women: <50 years old, ≤20 mm/h; >50 years old, ≤30 mm/h); N/A, not available; T‐DM1, trastuzumab emtansine.

^a^ The patient's colonoscopy and inflammatory markers were not performed on Day 28 as a result of treatment hold due to spinal fracture. Interim values, measured on Day 28 of treatment, were ESR 1.6 mg/L and CRP 38 mm/h.

**FIGURE 3:**
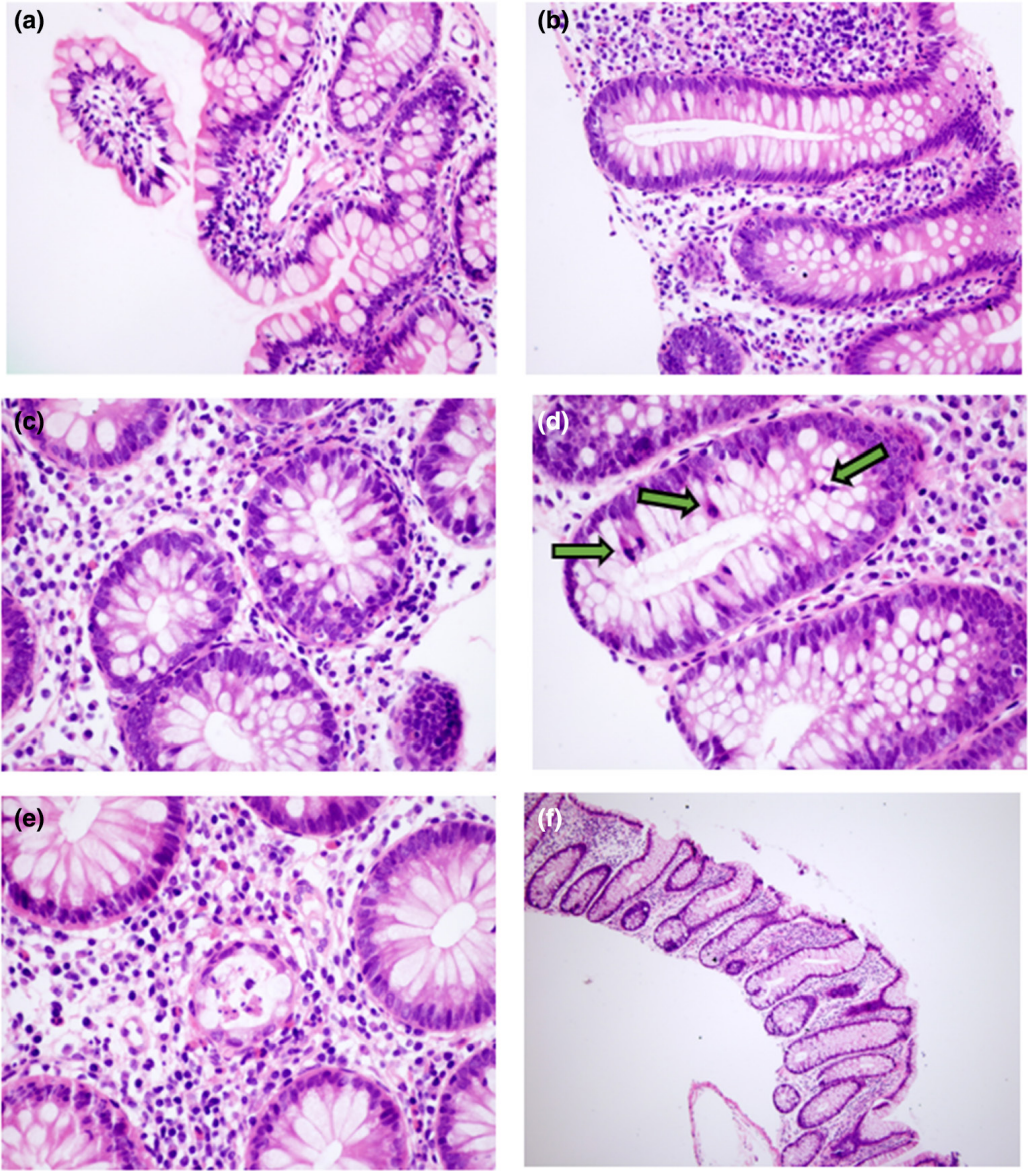
Intestinal changes in a patient who was treated with neratinib. Representative images of hematoxylin and eosin staining of patient 001 at the conclusion of cycle 1 are shown. (a) Cecum, 10× magnification; (b) cecum, 20× magnification; (c) ascending colon, 40× magnification; (d) proximal transverse colon, 40× magnification; green arrows indicate mitotic figures; (e) splenic flexure, 40× magnification; (f) splenic flexure, 4× magnification.

Patient 001 (age 45, postmenopausal, ECOG PS 0, MBC) was initially diagnosed with hormone receptor‐positive (HR+), and HER2‐positive (IHC 3+) invasive left breast carcinoma (stage cT3N+). Neoadjuvant chemotherapy was followed by radical mastectomy, radiotherapy, and adjuvant goserelin + tamoxifen + trastuzumab, then trastuzumab + tamoxifen. After development of cutaneous disease, she received first‐line docetaxel + pertuzumab + trastuzumab and second‐line trastuzumab emtansine (T‐DM1); she had a scant response to treatment and PD. The patient indicated she was CP prior to entering the study. Upon entering the present study, she received neratinib 240 mg/day in cycle 1 with no dose interruptions (treatment duration 113 days); capecitabine was added in cycle 2. The patient experienced diarrhea throughout treatment with neratinib (multiple grade 1 events). Biopsies taken during the second colonoscopy indicated focal nonspecific intraepithelial lymphocytes and capillary ectasia in the terminal ileum (Figure 3a), focal active colitis in the cecum (Figure 3b), mild focal active colitis in the ascending colon (Figure 3c), mitosis in the proximal transverse colon (Figure 3d), crypt branching and dropout in the splenic flexure (Figure 3e), and minimal nonspecific crypt branching in the sigmoid colon (Figure 3f).

The patient initially responded to neratinib treatment but experienced PD in cycle 5, at which point she began treatment with capecitabine + trastuzumab. She died 6 weeks later as a result of respiratory insufficiency caused by pleural effusion related to aggressive skin disease.

**FIGURE 4:**
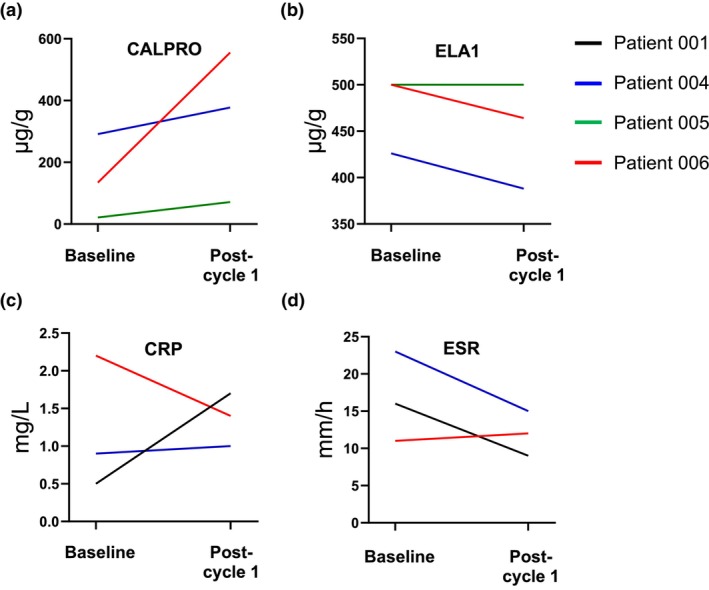
Changes in fecal calprotectin (a), fecal elastase (b), C‐reactive protein (c), and erythrocyte sedimentation rate (d) at baseline and after cycle 1. Data only shown if both timepoints were available.

Patient 004 (age 70, postmenopausal, ECOG PS 0, MBC) underwent mastectomy and lymph node biopsy for invasive carcinoma of the right breast. She was diagnosed with stage pT2N0(sn)M0, HR+, HER2+ disease and received adjuvant chemotherapy with 5‐FU + epirubicin + cyclophosphamide, followed by docetaxel + trastuzumab, then 15 cycles of trastuzumab and endocrine treatment with letrozole. Following right visceral pleural recurrence, she received paclitaxel + pertuzumab + trastuzumab and maintenance pertuzumab + trastuzumab + anastrozole. Pleural progression was treated with palliative T‐DM1; subsequent third‐line treatment consisted of vinorelbine + trastuzumab. After lung progression with lymphangitis was identified, the patient entered the study and received neratinib at 240 mg/day for 30 days, interrupted for 1 day before her first colonoscopy; capecitabine was initiated at the start of cycle 2. Neratinib treatment duration was 455 days overall. Treatment with neratinib resolved the lymphangitis and lung nodules. The patient stated she was CP before entering the study. No abnormal histopathological features were observed during the second colonoscopy, although nonspecific changes were noted in the terminal ileum, and nonspecific scattered intraepithelial eosinophils were observed in the ascending colon. Her fecal calprotectin levels increased from 291 to 377 μg/g between the first and second colonoscopies (Figure 4). Loperamide was administered as needed for continuous episodes of grade 1 diarrhea.

The patient subsequently had PD to the lung identified by CT scan and newly diagnosed bone disease on bone scan, but no brain metastasis on MRI. She subsequently underwent treatment with trastuzumab deruxtecan.

Patient 005 (age 40, premenopausal, ECOG PS 0, EBC) was initially diagnosed with cT2N0M0 BC (grade 3, ER 95%, PR 75%, HER2 3+, and Ki67 40%). After 5 months of tamoxifen + trastuzumab + pertuzumab, she had a mastectomy and sentinel node biopsy, followed by docetaxel + carboplatin + trastuzumab + pertuzumab, then trastuzumab + pertuzumab. She also received letrozole during this period. Prior to entering the study, the patient alluded to being DP. Upon entering the present study, she received extended adjuvant neratinib 240 mg/day in addition to letrozole and goserelin hormone therapy. Treatment was uninterrupted during the study period (treatment duration 361 days).

Neratinib was well tolerated, with one episode of diarrhea and two vomiting episodes, as well as non‐treatment−related headaches (all AEs were grade 1). The patient's fecal calprotectin levels increased from 21 to 71 μg/g over the observation period. Biopsy samples taken during the second colonoscopy revealed increased intraepithelial lymphocytes in the cecum and ascending colon, as well as patchy neutrophilic cryptitis in the proximal transverse colon.

Patient 006 (age 30, premenopausal, ECOG PS 0, MBC) was initially diagnosed with bilateral mammary carcinoma with large hepatic and lymph node metastases and bone metastases (stage IV, ER‐positive, PR‐positive, HER2 3+, Ki67 60%). She underwent vertebral radiotherapy and received 34 cycles of palliative docetaxel (10 months only) + trastuzumab + pertuzumab, in addition to goserelin and zoledronate and whole brain radiotherapy for brain metastases. Her best response was a partial response. Upon diagnosis of PD in the brain and bones, she was treated with T‐DM1 and had a partial response until CT scan identified retrosternal PD. Before the study began, the patient described herself as DP. She entered the present study and received neratinib 240 mg/day; neratinib was discontinued for 1 day prior to the second colonoscopy but was otherwise uninterrupted (treatment duration 325 days). Her best response was stable disease in the bone, liver, and lymph nodes. The patient left the study when she experienced brain progression. She began treatment with capecitabine + dexamethasone, but died as a result of central nervous system PD.

During the observation period, the patient experienced one episode of diarrhea and soft stools (treatment‐related; grade 1). Her fecal calprotectin levels increased from 134 to 555 μg/g between the first and second colonoscopies. No abnormal histopathological features were observed in the biopsy samples collected during the second colonoscopy.

### 3.3 Safety

The observed safety profile in this cohort of neratinib‐treated patients is consistent with the known safety profile of neratinib, and diarrhea was the most commonly observed AE as previously reported (Table S1). SAEs are described in Table S2.

## 4 DISCUSSION

Although neratinib has been shown to improve outcomes for patients with HER2+ BC, neratinib‐induced diarrhea can present challenges and must be actively managed with dose escalation and/or antidiarrheal prophylaxis, as well as dose reductions and dose holds. To date, confirmation of preclinical findings pertaining to the pathophysiology of neratinib‐induced diarrhea in neratinib‐treated HER2+ BC patients has remained elusive.

The preclinical findings described in this report reveal that colons from neratinib‐treated rats frequently showed degeneration, necrosis, and loss of lumen‐lining epithelium with migration of adjacent, viable enterocytes over the denuded surface in an attempt to maintain the integrity of the mucosal epithelial lining. In damaged glands, there was proliferation of enterocyte precursor cells in the crypt compartment, protective mucus extrusion by goblet cells, and an increased proprial and submucosal inflammatory infiltrate, mainly lymphocytes and eosinophils. These findings are consistent with earlier published reports in rat models that implied an inflammatory component of neratinib‐induced diarrhea (Secombe et al., 2019). The results of the preclinical study provided no evidence of loss of mucosal barrier function in the distal colon with neratinib, as similar resistance and FITC concentrations were observed in neratinib‐ and vehicle‐treated animals. To further interrogate epithelial barrier recovery processes, future work could examine expression of tight junction and other adherence proteins as potential markers of resistance to neratinib‐induced diarrhea, and additionally evaluate more proximal regions of the intestine. Likewise, there was no significant increase in secretory propensity in neratinib‐treated rats. Similar *I*_sc_ and responses to carbachol and forskolin were observed in both groups, contrasting with earlier work in healthy human volunteers that demonstrated fecal osmolar gap calculations consistent with secretory diarrhea following treatment with neratinib. However, the same study also identified inflammatory markers (fecal lactoferrin) in roughly 50% of the participants experiencing grade 2 diarrhea, further pointing to a considerable role of inflammation in neratinib‐mediated diarrhea (Abbas et al., 2012), but the regions of the intestine that contribute most to inflammation remain undetermined. Interestingly, a recent small study in patients treated with neratinib in the extended adjuvant setting demonstrated antidiarrheal activity of crofelemer, an oral, antisecretory antidiarrheal derived from the Amazonian *Croton lechleri* tree, additionally suggesting a secretory element of neratinib‐mediated diarrhea as seen in previous preclinical studies with TKIs (Jacob et al., 2023).

The phase 2 clinical portion of this work aimed at characterizing the colon pathogenesis of neratinib‐induced diarrhea in patients with HER2+ BC. Two patients, one with EBC and one with MBC, presented with mild pathological changes to their colon, whereas pathological changes were absent in the other two patients, both of whom had MBC. The histopathological changes in the former two patients included, but were not limited to, increases in infiltrating lymphocytes (i.e., in the cecum, ascending colon, and terminal ileum), patchy neutrophilic cryptitis in the proximal transverse colon, capillary ectasia in the terminal ileum, and focal active colitis in the cecum and ascending colon. Additionally, mitosis in the proximal transverse colon, crypt branching and dropout in the splenic flexure, and minimal nonspecific crypt branching in the sigmoid colon, were noted. The observations were broadly consistent with those in the rat model. Additionally, there was a rise in fecal calprotectin levels in all patients with available data, indicating gastrointestinal inflammation, even in the absence of any histopathological changes. However, no clear trend was observed regarding other markers of diarrhea and inflammation (CRP, ESR, and ELA1).

Although outside the scope of these studies, the gut microbiome may be an additional factor affecting neratinib‐mediated diarrhea (Secombe et al., 2020). Recently, the narrow‐spectrum antibiotics vancomycin and neomycin were shown to reduce the incidence of neratinib‐induced diarrhea in the rat model (Secombe et al., 2022). In that model, neratinib treatment was associated with increases in the relative abundance of the family *Ruminococcaceae* and the genus *Oscillospira* and relative abundance reductions in levels of the genus *Blautia*. In particular, neomycin increased *Blautia*'s relative abundance, thus providing further evidence of an association between diarrhea and microbiome changes (Secombe et al., 2021).

Potential treatments aimed at mitigating treatment‐induced diarrhea have been investigated in vivo. The locally acting steroid budesonide, which is used in the treatment of gastrointestinal conditions, and colesevelam, a bile acid sequestrant, have been shown to be effective interventions in the rat model of neratinib‐induced diarrhea (Secombe et al., 2019). The latter intervention was based on the actuality that ileal inflammation is associated with bile‐acid malabsorption (Stelzner et al., 2001). Based on these findings, the CONTROL trial (NCT02400476) was undertaken to assess the effects of several antidiarrheal strategies on the occurrence of grade ≥3 diarrhea (Barcenas et al., 2020; Chan et al., 2023). Patients were enrolled sequentially into six different cohorts and received daily neratinib for 1 year after trastuzumab‐based adjuvant therapy. The six cohorts evaluated prophylaxis with loperamide, budesonide, or colestipol, and also investigated dose escalation of neratinib. CONTROL established that the most effective way to mitigate the occurrence of grade ≥3 diarrhea was a dose‐escalation regimen combined with loperamide as needed, starting at 120 mg QD of neratinib and gradually increasing to 240 mg QD over the course of 2 weeks (Chan et al., 2023). In this dose‐escalation cohort, grade ≥3 diarrhea rates and treatment discontinuations of 13.3% and 3.3%, respectively, were noted, which are lower than historical diarrhea and discontinuation rates in the ExteNET study (Chan et al., 2016). A series of case reports from a real‐world clinical setting underscored that neratinib‐associated diarrhea can be effectively managed by dose escalation and prophylactic use of antidiarrheals (Kruse et al., 2022). Moreover, the ELEANOR study has shown that diarrhea prophylaxis and dose‐escalation strategies markedly improved neratinib treatment tolerability in a routine clinical setting (Bartsch et al., 2023).

Limitations of the current work should be considered. The preclinical study design did not enable probing of sodium absorption dysfunction due to the use of amiloride in the Ussing chamber setup, which may be impacted by neratinib itself and concurrent inflammation. The clinical study comprised a small number of participants and was not equally balanced regarding disease stage, with only one of four evaluable patients treated in the curative setting. Concomitant use of medications implicated in causing microscopic colitis was prohibited; however, patients in the late‐stage setting had been exposed to a multitude of other agents, which might have had an impact on their colonic mucosa. Additionally, the second colonoscopy was performed after 28 days of neratinib monotherapy and did not coincide with any acute diarrhea episodes, and thus transient pathological changes may have been missed. Most importantly, patients in the clinical study received loperamide antidiarrheal prophylaxis, potentially explaining the low‐grade diarrhea observed throughout. Patients with more severe diarrhea episodes might show exacerbated pathological changes to their colonic mucosa compared with those observed in the current study population. Hence, further evaluation in a patient population with higher‐grade diarrhea is warranted.

## 5 CONCLUSION

This report is the first to investigate histopathological changes mediated by neratinib‐induced diarrhea in a rat model of diarrhea as well as in patients with HER2‐positive BC of different stages. Among the multifactorial etiologies that have been suggested for neratinib‐induced diarrhea, the current data shown support the role of an inflammatory component in this condition. Our findings could therefore help devise additional diarrhea mitigation strategies (i.e., the use of anti‐inflammatory agents other than budesonide) to complement the effective dose‐escalation strategy identified for neratinib in the CONTROL trial.

## AUTHOR CONTRIBUTIONS

J.B., S.B., V.D.Z., J.F., and J.A.D.P. conceived and designed research; J.B., S.B., V.D.Z., J.F., D.D., A.W., G.F.B., and J.A.D.P. performed experiments, analyzed data, interpreted results of experiments; J.B., S.B., V.D.Z., J.F., D.D., G.F.B., and J.A.D.P. prepared figures; J.B., S.B., V.D.Z., J.F., D.D., B.C., G.F.B., A.W., and J.A.D.P. drafted manuscript; J.B., S.B., V.D.Z., J.F., D.D., B.C., G.F.B., A.W., and J.A.D.P. edited and revised manuscript; J.B., S.B., V.D.Z., J.F., D.D., B.C., G.F.B., A.W., and J.A.D.P. approved final version of manuscript.

## FUNDING INFORMATION

Puma Biotechnology, Inc. sponsored the phase II trial described herein and also funded the provision of editorial/writing support provided by Miller Medical Communications Ltd. The preclinical studies led by J.B. were supported by research funding from Puma Biotechnology, Inc.

## DISCLOSURES

Daniel DiPrimeo, Blaire Cooke, Georg F Bischof, and Alvin Wong: Full‐time employment with Puma Biotechnology, Inc. Ownership interest (stock, stock options) in Puma Biotechnology, Inc.

## ETHICS STATEMENT

The preclinical study was approved by the Animal Ethics Committee of the University of Adelaide (study number M‐2019‐025) and complied with the National Health and Medical Research Council (Australia) Code of Practice for Animal Care in Research and Training (2013). The clinical study was approved by the Ethics Committee for Clinical Research (CEIC) and the National Authority for Medicines and Health Products, I.P. (INFARMED) in Portugal (CEIC Code: 20190900, decision number 48/VPCD/2020). The study was carried out in accordance with the current revised version of the Declaration of Helsinki and Good Clinical Practice and other applicable legislation. All patients provided written informed consent.

## Supporting information


Data S1.

## Data Availability

The authors declare that the data supporting the findings of this study are available within the article. Qualified researchers and study participants may submit requests for other study documentation and clinical trial data to clinicaltrials@pumabiotechnology.com for consideration.

## References

[phy270008-bib-0001] Abbas, R. , Hug, B. A. , Leister, C. , & Sonnichsen, D. (2012). A double‐blind, randomized, multiple‐dose, parallel‐group study to characterize the occurrence of diarrhea following two different dosing regimens of neratinib, an irreversible pan‐ErbB receptor tyrosine kinase inhibitor. Cancer Chemotherapy and Pharmacology, 70, 191–199. 22418773 10.1007/s00280-012-1857-3

[phy270008-bib-0002] Awada, A. , Colomer, R. , Inoue, K. , Bondarenko, I. , Badwe, R. A. , Demetriou, G. , Lee, S. C. , Mehta, A. O. , Kim, S. B. , Bachelot, T. , Goswami, C. , Deo, S. , Bose, R. , Wong, A. , Xu, F. , Yao, B. , Bryce, R. , & Carey, L. A. (2016). Neratinib plus paclitaxel vs trastuzumab plus paclitaxel in previously untreated metastatic ERBB2‐positive breast cancer: The NEfERT‐T randomized clinical trial. JAMA Oncology, 2, 1557–1564. 27078022 10.1001/jamaoncol.2016.0237

[phy270008-bib-0003] Barcenas, C. H. , Hurvitz, S. A. , Di Palma, J. A. , Bose, R. , Chien, A. J. , Iannotti, N. , Marx, G. , Brufsky, A. , Litvak, A. , Ibrahim, E. , Alvarez, R. H. , Ruiz‐Borrego, M. , Chan, N. , Manalo, Y. , Kellum, A. , Trudeau, M. , Thirlwell, M. , Garcia Saenz, J. , Hunt, D. , … Chan, A. (2020). Improved tolerability of neratinib in patients with HER2‐positive early‐stage breast cancer: The CONTROL trial. Annals of Oncology, 31, 1223–1230. 32464281 10.1016/j.annonc.2020.05.012

[phy270008-bib-0004] Bartsch, R. , Harbeck, N. , Wrobel, D. , Zaiss, M. , Terhaag, J. , Guth, D. , Distelrath, A. , Wuerstlein, R. , Zahn, M.‐O. , Lüftner, D. , Schwitter, M. , Balic, M. , Jackisch, C. , Müller, V. , Rinnerthaler, G. , Schmidt, M. , Zaman, K. , Schinköthe, T. , Resch, A. , & Breitenstein, U. (2023). Abstract P2‐01‐01: Interim analysis (*n*=200) from ELEANOR: A multi‐national, prospective, non‐interventional study among patients with HER2+ and HR+ early breast cancer treated with extended adjuvant neratinib in the clinical routine. Cancer Research, 83, P2.

[phy270008-bib-0005] Bowen, J. M. , Mayo, B. J. , Plews, E. , Bateman, E. , Stringer, A. M. , Boyle, F. M. , Finnie, J. W. , & Keefe, D. M. K. (2012). Development of a rat model of oral small molecule receptor tyrosine kinase inhibitor‐induced diarrhea. Cancer Biology & Therapy, 13, 1269–1275. 22895076 10.4161/cbt.21783 PMC3493434

[phy270008-bib-0006] Chan, A. , Delaloge, S. , Holmes, F. A. , Moy, B. , Iwata, H. , Harvey, V. J. , Robert, N. J. , Silovski, T. , Gokmen, E. , Von Minckwitz, G. , Ejlertsen, B. , Chia, S. K. , Mansi, J. , Barrios, C. H. , Gnant, M. , Buyse, M. , Gore, I. , Smith, J., 2nd , Harker, G. , … Martin, M. (2016). Neratinib after trastuzumab‐based adjuvant therapy in patients with HER2‐positive breast cancer (ExteNET): A multicentre, randomised, double‐blind, placebo‐controlled, phase 3 trial. Lancet Oncology, 17, 367–377. 26874901 10.1016/S1470-2045(15)00551-3

[phy270008-bib-0007] Chan, A. , Ruiz‐Borrego, M. , Marx, G. , Chien, A. J. , Rugo, H. S. , Brufsky, A. , Thirlwell, M. , Trudeau, M. , Bose, R. , García‐Sáenz, J. A. , Egle, D. , Pistilli, B. , Wassermann, J. , Cheong, K. A. , Schnappauf, B. , Semsek, D. , Singer, C. F. , Foruzan, N. , Diprimeo, D. , … Barcenas, C. H. (2023). Final findings from the CONTROL trial: Strategies to reduce the incidence and severity of neratinib‐associated diarrhea in patients with HER2‐positive early‐stage breast cancer. Breast, 67, 94–101. 36702070 10.1016/j.breast.2022.12.003 PMC9982309

[phy270008-bib-0009] European Medicines Agency . (2022). Neratinib Summary of product characteristics. https://www.medicines.org.uk/emc/product/10477/smpc#gref

[phy270008-bib-0010] Food & Drug Administration . (2022). Nerlynx highlights of prescribing information. https://nerlynxhcp.com/pdf/full‐prescribing‐information.pdf

[phy270008-bib-0011] Jacob, S. , Johnson, M. , Roque, B. , Quintal, L. , Rugo, H. S. , Melisko, M. , & Chien, A. J. (2023). Crofelemer for the management of neratinib‐associated diarrhea in patients with HER2+ early‐stage breast cancer. Clinical Breast Cancer, 23, 721–728. 37474374 10.1016/j.clbc.2023.06.014

[phy270008-bib-0012] Kruse, M. L. , Kang, I. M. , Bagegni, N. A. , Howell, W. T. , Moore, H. C. F. , Bedell, C. H. , & Stokoe, C. T. (2022). Management of diarrhea in patients with HER2‐positive breast cancer treated with neratinib: A case series and summary of the literature. Oncology and Therapy, 10, 279–289. 34800263 10.1007/s40487-021-00178-w PMC8605449

[phy270008-bib-0013] Le Du, F. , Diéras, V. , & Curigliano, G. (2021). The role of tyrosine kinase inhibitors in the treatment of HER2+ metastatic breast cancer. European Journal of Cancer, 154, 175–189. 34280871 10.1016/j.ejca.2021.06.026

[phy270008-bib-0014] Martin, M. , Holmes, F. A. , Ejlertsen, B. , Delaloge, S. , Moy, B. , Iwata, H. , Von Minckwitz, G. , Chia, S. K. L. , Mansi, J. , Barrios, C. H. , Gnant, M. , Tomasevic, Z. , Denduluri, N. , Separovic, R. , Gokmen, E. , Bashford, A. , Ruiz Borrego, M. , Kim, S. B. , Jakobsen, E. H. , … Chan, A. (2017). Neratinib after trastuzumab‐based adjuvant therapy in HER2‐positive breast cancer (ExteNET): 5‐year analysis of a randomised, double‐blind, placebo‐controlled, phase 3 trial. The Lancet Oncology, 18, 1688–1700. 29146401 10.1016/S1470-2045(17)30717-9

[phy270008-bib-0015] Mortimer, J. , Di Palma, J. , Schmid, K. , Ye, Y. , & Jahanzeb, M. (2019). Patterns of occurrence and implications of neratinib‐associated diarrhea in patients with HER2‐positive breast cancer: Analyses from the randomized phase III ExteNET trial. Breast Cancer Research, 21, 32. 30813966 10.1186/s13058-019-1112-5 PMC6391844

[phy270008-bib-0017] National Comprehensive Cancer Network . (2024). Clinical Practice Guidelines in Oncology. Breast Cancer. Version 4. https://www.nccn.org/professionals/physician_gls/pdf/breast.pdf

[phy270008-bib-0018] Saura, C. , Oliveira, M. , Feng, Y.‐H. , Dai, M.‐S. , Hurvitz, S. A. , Kim, S.‐B. , Moy, B. , Delaloge, S. , Gradishar, W. J. , Masuda, N. , Palacova, M. , Trudeau, M. E. , Mattson, J. , Yap, Y. S. , Bryce, R. , Yao, B. , Bebchuk, J. D. , Keyvanjah, K. , Brufsky, A. , & Investigators, N. (2019). Neratinib + capecitabine versus lapatinib + capecitabine in patients with HER2+ metastatic breast cancer previously treated with ≥2 HER2‐directed regimens: Findings from the multinational, randomized, phase III NALA trial. Journal of Clinical Oncology, 37, 1002. 10.1200/JCO.20.00147 PMC7499616 32678716

[phy270008-bib-0019] Secombe, K. R. , Ball, I. A. , Shirren, J. , Wignall, A. D. , Finnie, J. , Keefe, D. , Avogadri‐Connors, F. , Olek, E. , Martin, D. , Moran, S. , & Bowen, J. M. (2019). Targeting neratinib‐induced diarrhea with budesonide and colesevelam in a rat model. Cancer Chemotherapy and Pharmacology, 83, 531–543. 30535958 10.1007/s00280-018-3756-8

[phy270008-bib-0020] Secombe, K. R. , Ball, I. A. , Shirren, J. , Wignall, A. D. , Keefe, D. M. , & Bowen, J. M. (2021). Pathophysiology of neratinib‐induced diarrhea in male and female rats: Microbial alterations a potential determinant. Breast Cancer, 28, 99–109. 32683606 10.1007/s12282-020-01133-9

[phy270008-bib-0021] Secombe, K. R. , Ball, I. A. , Wignall, A. D. , Bateman, E. , Keefe, D. M. , & Bowen, J. M. (2022). Antibiotic treatment targeting gram negative bacteria prevents neratinib‐induced diarrhea in rats. Neoplasia, 30, 100806. 35561424 10.1016/j.neo.2022.100806 PMC9111977

[phy270008-bib-0022] Secombe, K. R. , Van Sebille, Y. Z. A. , Mayo, B. J. , Coller, J. K. , Gibson, R. J. , & Bowen, J. M. (2020). Diarrhea induced by small molecule tyrosine kinase inhibitors compared with chemotherapy: Potential role of the microbiome. Integrative Cancer Therapies, 19, 1534735420928493. 32493068 10.1177/1534735420928493 PMC7273583

[phy270008-bib-0023] Stelzner, M. , Somasundaram, S. , & Khakberdiev, T. (2001). Systemic effects of acute terminal ileitis on uninflamed gut aggravate bile acid malabsorption. Journal of Surgical Research, 99, 359–364. 11469911 10.1006/jsre.2001.6137

[phy270008-bib-0024] Van Sebille, Y. Z. A. , Gibson, R. J. , Wardill, H. R. , Ball, I. A. , Keefe, D. M. K. , & Bowen, J. M. (2018). Dacomitinib‐induced diarrhea: Targeting chloride secretion with crofelemer. International Journal of Cancer, 142, 369–380. 28921512 10.1002/ijc.31048

[phy270008-bib-0025] Van Sebille, Y. Z. A. , Gibson, R. J. , Wardill, H. R. , Secombe, K. R. , Ball, I. A. , Keefe, D. M. K. , Finnie, J. W. , & Bowen, J. M. (2017). Dacomitinib‐induced diarrhoea is associated with altered gastrointestinal permeability and disruption in ileal histology in rats. International Journal of Cancer, 140, 2820–2829. 28316082 10.1002/ijc.30699

