# Supplementary material for: Preclinical and clinical evaluation through serial colonoscopic evaluation of neratinib‐induced diarrhea in HER2‐positive breast cancer—A pilot study
Source: Physiol Rep. 2024 Aug 26;12(16):e70008. doi: 10.14814/phy2.70008 (PMC11347019; doi:10.14814/phy2.70008)
Supplement: Supplementary file 1 — Data S1. [file PHY2-12-e70008-s001.docx]

# Supplemental information

### Inclusion criteria

Each patient will be entered into this study only if she/he meets all of the following criteria:

1. Aged ≥18 years at signing of informed consent.

2. Histologically confirmed stage 1 through stage 4 primary adenocarcinoma of the breast (Edge and Compton, 2010).

3. Documented HER2 overexpression or gene-amplified tumor by a validated approved method (Wolff et al., 2013).

4. Patients with confirmed stage 1 to 3c BC receiving neratinib monotherapy must have completed a course of prior adjuvant trastuzumab or experienced side effects that resulted in early discontinuation of trastuzumab that have since resolved.

5. Patients with mBC must have had at least two prior HER2-directed regimens.

6. Left ventricular ejection fraction (LVEF) ≥50% measured by multiple-gated acquisition scan or echocardiogram.

7. ECOG status of 0 to 1.

8. Negative β-human chorionic gonadotropin pregnancy test for premenopausal women of reproductive capacity (those who are biologically capable of having children) and for women less than 12 months after menopause. Women are considered postmenopausal if they are ≥12 months without menses, in the absence of endocrine or anti-endocrine therapies.

9. Women of childbearing potential must agree and commit to the use of a highly effective non-hormonal method of contraception, i.e., intrauterine device, bilateral tubal ligation, vasectomized partner, or abstinence (only when it is the preferred lifestyle of the patient), from the time of informed consent until 28 days after the last dose of the investigational products. Men (male patient) with a female partner of childbearing potential must agree and commit to use condom and the female partner must agree and commit to use a highly effective method of contraception (i.e., any of the above methods, or for females, hormonal contraception associated with inhibition of ovulation) while on treatment and for 3 months after last dose of investigational products.

10. Recovery (i.e., to Grade 1 or baseline) from all clinically significant AEs related to prior therapies (excluding alopecia, neuropathy, and nail changes).

11. No major bleeding diathesis or use of anticoagulants that would pose a high risk for endoscopic procedure.

12. Provide written, informed consent to participate in the study and follow the study procedures.

### Exclusion criteria

1. Patients with confirmed stage 1 through stage 3c BC currently receiving chemotherapy, radiation therapy, immunotherapy, or biotherapy for BC.

2. Patients with MBC who have received prior capecitabine or HER2 directed TKI therapy.

3. Currently using drugs that have been implicated as causing microscopic colitis/watery diarrhea, such as acarbose, aspirin, proton pump inhibitors, nonsteroidal anti-inflammatory drugs (NSAIDs), histamine H2-receptor antagonists, selective serotonin reuptake inhibitors, and ticlopidine (Pardi, 2017).

4. Major surgery within <28 days of starting treatment or received chemotherapy, investigational agents, or other cancer therapy, except hormonal therapy (e.g., tamoxifen, aromatase inhibitors), <14 days prior to the initiation of investigational products.

5. Active uncontrolled cardiac disease, including cardiomyopathy, congestive heart failure (New York Heart Association functional classification of ≥2; including individuals who currently use digitalis, beta-blockers, or calcium channel blockers specifically for congestive heart failure), unstable angina, myocardial infarction within 12 months of enrollment, or ventricular arrhythmia.

6. QTc interval >0.450 seconds (males) or >0.470 (females), or known history of QTc prolongation or Torsade de Pointes.

7. Diagnosis of inflammatory bowel disease.

8. Screening laboratory assessments outside the following limits:

1. Absolute neutrophil count: ≤1000/µl (≤1.0 x 10^9^/L);
2. Platelet count: ≤50,000/µl (≤100 x 10^9^/L);
3. Hemoglobin: ≤8 g/dL (transfusions allowed). Transfusions must be ≥14 days prior to initiation of treatment;
4. Total bilirubin: >1.5 x institutional upper limit of normal (ULN) (in case of known Gilbert’s syndrome, <2 x ULN is allowed)
5. Aspartate aminotransferase (AST) and/or alanine aminotransferase (ALT): >2.5 x institutional ULN (>5 x ULN if liver metastases are present);
6. Creatinine: creatinine clearance <30 mL/min (as calculated by Cockcroft-Gault formula or Modification of Diet in Renal Disease formula)
7. International Normalized Ratio: >1.5.

9. Active, unresolved infections.

10. Patients with a second malignancy, other than adequately treated non-melanoma skin cancers, in situ melanoma or in situ cervical cancer. Patients with other non-mammary malignancies must have been disease-free for at least 5 years.

11. Currently pregnant or breast-feeding.

12. Significant chronic gastrointestinal disorder with diarrhea as a major symptom (eg, Crohn’s disease, malabsorption, or Grade ≥2 NCI CTCAE v.4.0 diarrhea of any etiology at baseline).

13. Clinically active infection with hepatitis B or hepatitis C virus.

14. Evidence of significant medical illness, abnormal laboratory finding, or psychiatric illness/social situations that could, in the Investigator’s judgment, make the patient inappropriate for this study.

15. Known hypersensitivity to any component of the investigational products; known allergies to any of the medications or components of medications used in the trial.

16. Unable or unwilling to swallow tablets.

## Supplemental Table 1. Treatment-emergent adverse events in Cycle 1 and post 28 days

| Adverse event, n (%) | Cycle 1 (*n* = 5) | | |  | Post 28 days (*n* = 4)^a^ | | |
| --- | --- | --- | --- | --- | --- | --- | --- |
|  | grade 1 | grade 2 | grade ≥3 |  | grade 1 | grade 2 | grade ≥3 |
| Diarrhea | 4 (80) | 1 (20) | 0 |  | 3 (75) | 0 | 0 |
| Vomiting | 3 (60) | 0 | 0 |  | 0 | 0 | 0 |
| Abdominal distension | 1 (20) | 0 | 0 |  | 0 | 0 | 0 |
| Abdominal pain | 0 | 0 | 0 |  | 1 (25) | 0 | 0 |
| Constipation | 1 (20) | 0 | 0 |  | 0 | 0 | 0 |
| Flatulence | 1 (20) | 0 | 0 |  | 0 | 0 | 0 |
| Nausea | 1 (20) | 1 (20) | 0 |  | 0 | 0 | 0 |
| Paronychia | 1 (20) | 0 | 0 |  | 0 | 0 | 0 |
| Muscle spasm | 1 (20) | 0 | 0 |  | 0 | 0 | 0 |
| Headache | 1 (20) | 0 | 0 |  | 1 (25) | 0 | 0 |
| Pleural effusion | 0 | 0 | 1 (20) |  | 0 | 0 | 0 |
| Pericardial effusion | 0 | 0 | 1 (20) |  | 0 | 0 | 0 |
| Feces hard | 0 | 0 | 0 |  | 1 (25) | 0 | 0 |
| Feces soft | 0 | 0 | 0 |  | 1 (25) | 0 | 0 |
| Pulmonary infection | 0 | 0 | 0 |  | 0 | 0 | 1 (25) |
| Thrombocytopenia | 0 | 0 | 0 |  | 1 (25) | 0 | 0 |
| Dizziness | 0 | 0 | 0 |  | 1 (25) | 0 | 0 |

^a^From day 1 of Cycle 1 to 28 days after the end of Cycle 2

## Supplemental Table 2. Treatment-emergent serious adverse events in cycle 1 and post 28 days

| Event, n (%) | Cycle 1 (*n* = 5) | Post 28 days (*n* = 4) |
| --- | --- | --- |
| Pericardial effusion | 1 (20) | 0 |
| Pleural effusion | 1 (20) | 0 |
| Pulmonary infection | 0 | 1 (25) |

**Supplemental Figure 1. CONSORT diagram**


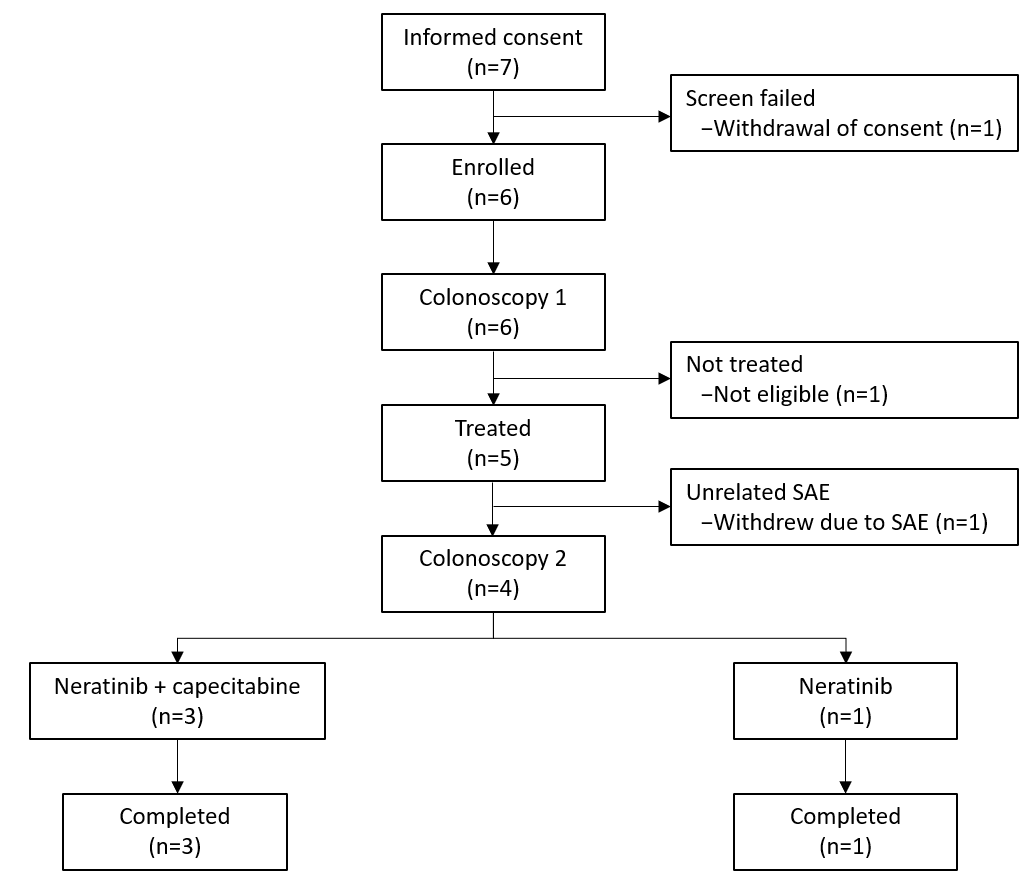


References

Edge, S. B., & Compton, C. C. (2010). The American Joint Committee on cancer: The 7th edition of the AJCC cancer staging manual and the future of TNM. *Annals of Surgical Oncology*, *17*, 1471–1474.

Pardi, D. S. (2017). Diagnosis and management of microscopic colitis. *American Journal of Gastroenterology*, *112*, 78–85.

Wolff, A. C., Hammond, M. E., Hicks, D. G., Dowsett, M., Mcshane, L. M., Allison, K. H., Allred, D. C., Bartlett, J. M., Bilous, M., Fitzgibbons, P., Hanna, W., Jenkins, R. B., Mangu, P. B., Paik, S., Perez, E. A., Press, M. F., Spears, P. A., Vance, G. H., Viale, G., & Hayes, D. F. (2013). Recommendations for human epidermal growth factor receptor 2 testing in breast cancer: American Society of Clinical Oncology/College of American Pathologists Clinical Practice Guideline Update. *Journal of Clinical Oncology*, *31*, 3997–4013.
